# The potential role of the pseudobranch of molly fish (*Poecilia sphenops*) in immunity and cell regeneration

**DOI:** 10.1038/s41598-023-34044-8

**Published:** 2023-05-29

**Authors:** Doaa M. Mokhtar, Ramy K. A. Sayed, Giacomo Zaccone, Alessio Alesci, Marwa M. Hussein

**Affiliations:** 1 Department of Cell and Tissues, Faculty of Veterinary Medicine, Assiut University, Assiut, 71526 Egypt; 2 Department of Anatomy and Embryology, Faculty of Veterinary Medicine, Sohag University, Sohag, 82524 Egypt; 3 Department of Veterinary Sciences, Polo Universitario dell’Annunziata, University of Messina, 98168 Messina, Italy; 4 Department of Chemical, Biological, Pharmaceutical and Environmental Sciences, University of Messina, 98168 Messina, Italy

**Keywords:** Cell biology, Developmental biology, Physiology, Structural biology, Zoology

## Abstract

The pseudobranch is a gill-like structure that exhibits great variations in structure and function among fish species, and therefore, it has remained a topic of investigation for a long time. This study was conducted on adult Molly fish (*Poecilia sphenops*) to investigate the potential functions of their pseudobranch using histological, histochemical, immunohistochemical analysis, and scanning electron microscopy. The pseudobranch of Molly fish was of embedded type. It comprised many rows of parallel lamellae that were fused completely throughout their length by a thin connective tissue. These lamellae consisted of a central blood capillary, surrounded by large secretory pseudobranch cells (PSCs). Immunohistochemical analysis revealed the expression of PSCs for CD3, CD45, iNOS-2, and NF-κB, confirming their role in immunity. Furthermore, T-lymphocytes-positive CD3, leucocytes-positive CD45, and dendritic cells-positive CD-8 and macrophage- positive APG-5 could be distinguished. Moreover, myogenin and TGF-β-positive PSCs were identified, in addition to nests of stem cells- positive SOX-9 were detected. Melanocytes, telocytes, and GFAP-positive astrocytes were also demonstrated. Scanning electron microscopy revealed that the PSCs were covered by microridges, which may increase the surface area for ionic exchange. In conclusion, pseudobranch is a highly specialized structure that may be involved in immune response, ion transport, acid–base balance, as well as cell proliferation and regeneration.

## Introduction

The pseudobranch is a red, gill-like structure that originates from the first gill arch and is attached to the internal surface of the operculum. It exhibits variation in form, size, and location in different fish species. Therefore, it has remained an aim of many studies for a long time^1^. The pseudobranch has a direct vascular connection with the choroid rete of the eye. The pseudobranch and the choroid rete appear to have a role in the maintenance of blood pressure of the eye through the elevation of the arterial oxygen pressure by enzymatically acidifying the arterial blood through the action of carbonic anhydrase^2,3^.

Pseudobranchs are present in most fish species, except for all species of the genera Gymnarchus and Cobitis, as well as a few species of the order Anguilliformes, and suborder Siluroidei^4^. Previous studies have reported several functions of the pseudobranch, including respiration, vision, osmoregulation, and endocrine functions^1^, as well as ocular oxygen secretion^5^ and sensory or chemoreceptors^6^. Moreover, pseudobranch cells (PSCs) are involved in blood acid–base balance and the production of carbonic anhydrase^7^.

*Poecilia sphenops* belongs to the genus *Poecilia* and is commonly known Molly. They inhabit freshwater streams and can also survive in the coastal brackish and marine waters of Mexico. Molly is considered one of the most popular feeder fish due to its high growth rate, brood number, birth size, and reproduction^8,9^.

Several previous studies reported the immune role of the gills^10–12^. Furthermore, our recent study on the gills of molly fish revealed that the gills contribute to fish immune reaction^13^. Interestingly, an individual report has indicated the role of pseudobranch in the fish’s immune reaction^14^. With the anatomical location of the pseudobranch beside the gills, in addition to its anatomical resemblance to the gill filaments and its originality from the mandibular gill arch^15^, it was hypothesized that this vital organ may have an immune role. The available literature lacks the histological and ultrastructural description of the pseudobranch; therefore, the motivation to conduct this study was to characterize the structure of pseudobranch of Molly fish with highlighting the potential role of its cellular constituents in immunity using histological and immunohistochemical analysis, as well as scanning electron microscopy. This topography of the pseudobranch will be considered to provide an interesting view for advanced physiological studies.

## Results

### Histological analysis

**Figure 1:**
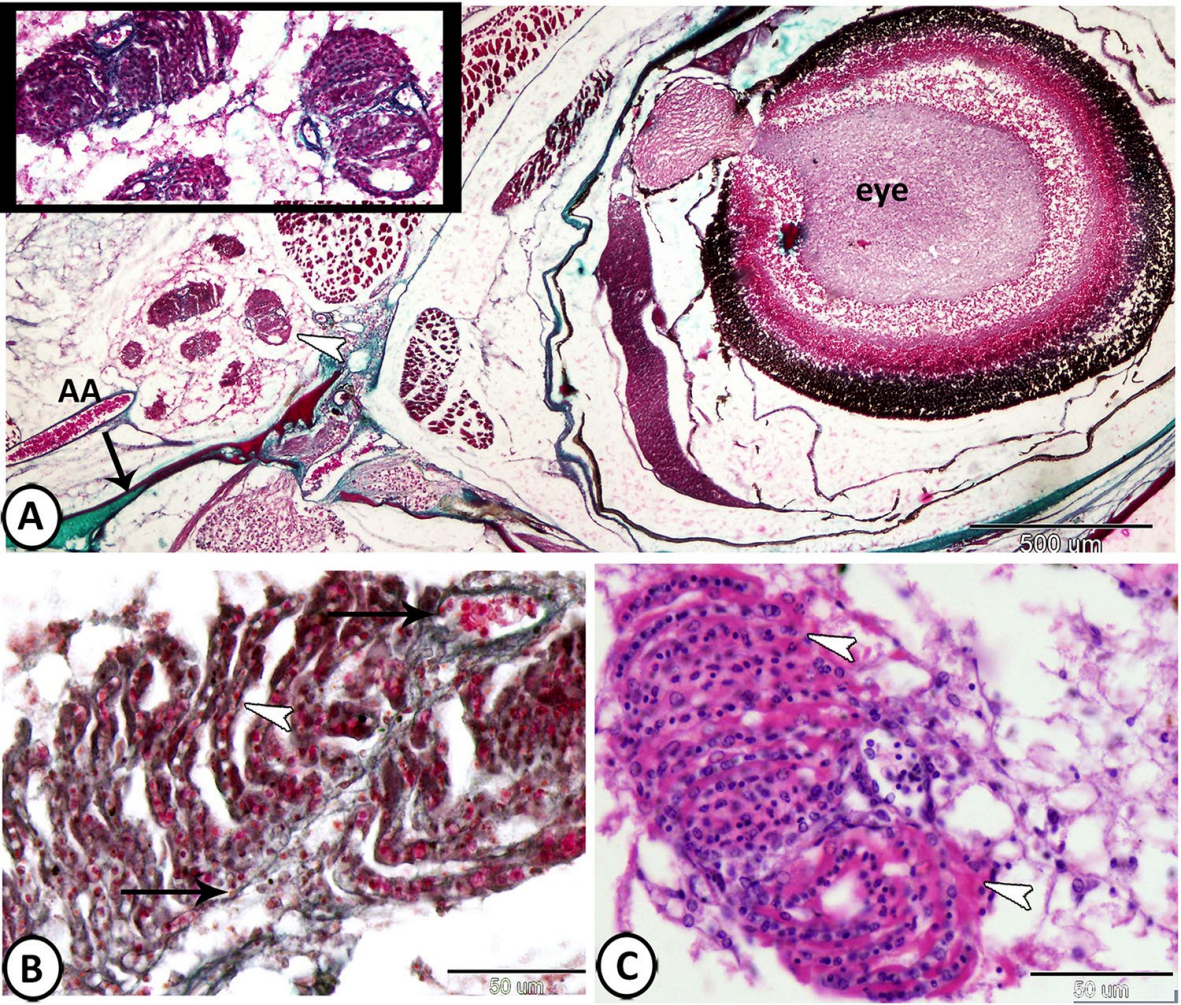
Histological analysis of the pseudobranch of Molly fish. (**A**) The pseudobranch (arrowhead, inserted image) is situated in the opercular cavity (arrow) and supplied with an afferent artery (AA) (Crossmon's trichrome). (**B**) The lamellae consist of a central blood capillary (arrows), which was surrounded by pseudobranch cells (arrowhead) (Crossmon's trichrome). (**C**) The lamellae comprise several rows of parallel lamellae (arrowheads) that are fused by a thin connective tissue (HE).

The pseudobranch of Molly fish was embedded in the connective tissues situated in the opercular cavity near the first gill arch and was supplied with an afferent artery. It exhibited the appearance of gill-like lamellae that were oriented in 6–7 lobules surrounded by a thin connective tissue (Fig. 1A). Its lamellae showed no contact with the external environment. The lamellae comprised several rows of parallel lamellae, which were fused to each other by a thin connective tissue throughout their length. The lamellae consisted of a central blood capillary, which was surrounded by large glandular pseudobranch cells (PSCs) (Fig. 1B,C).

**Figure 2:**
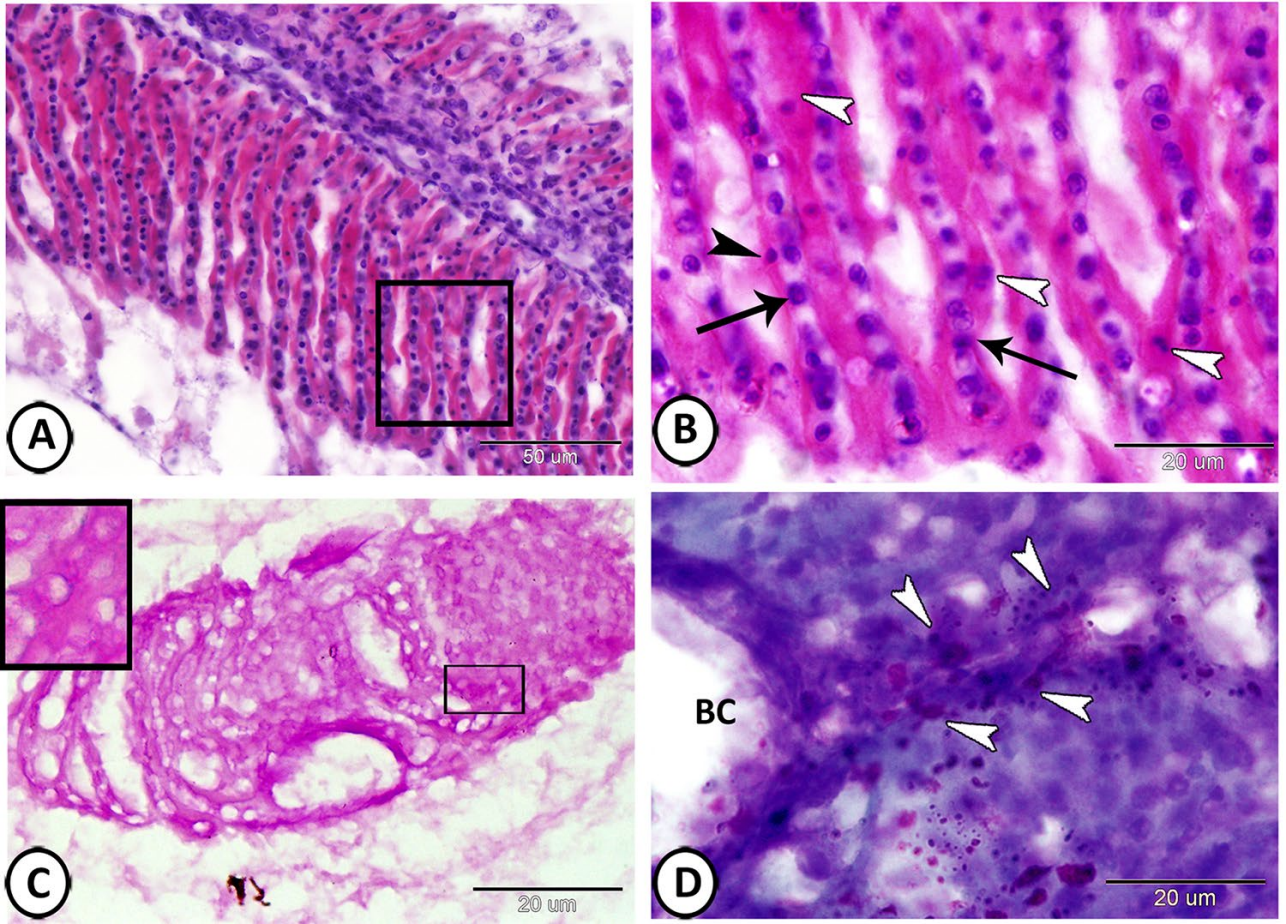
Histochemical analysis of the pseudobranch of Molly fish. (**A**,**B**) The PSCs (white arrowheads) are situated beneath the pavement cells (black arrowhead), separated by pillar cells (arrows) (HE). (**C**) The PSCs (boxed areas) show a positive reaction to PAS. (**D**) Many immune cells (arrowheads) could be demonstrated near the blood capillaries (BC) (Giemsa's stain).

The PSCs were situated beneath the pavement cells along the lamellae and were associated with the blood capillaries separated by pillar cells. The PSCs possessed acidophilic cytoplasm and a large centrally located nucleus (Fig. 2A,B). These cells showed a positive reaction for PAS (Fig. 2C). Many immune cells could be observed between the PSCs near the blood capillaries (Fig. 2D). No chloride cells could be observed in the pseudobranch of Molly fish.

### Immunohistochemical analysis

**Figure 3:**
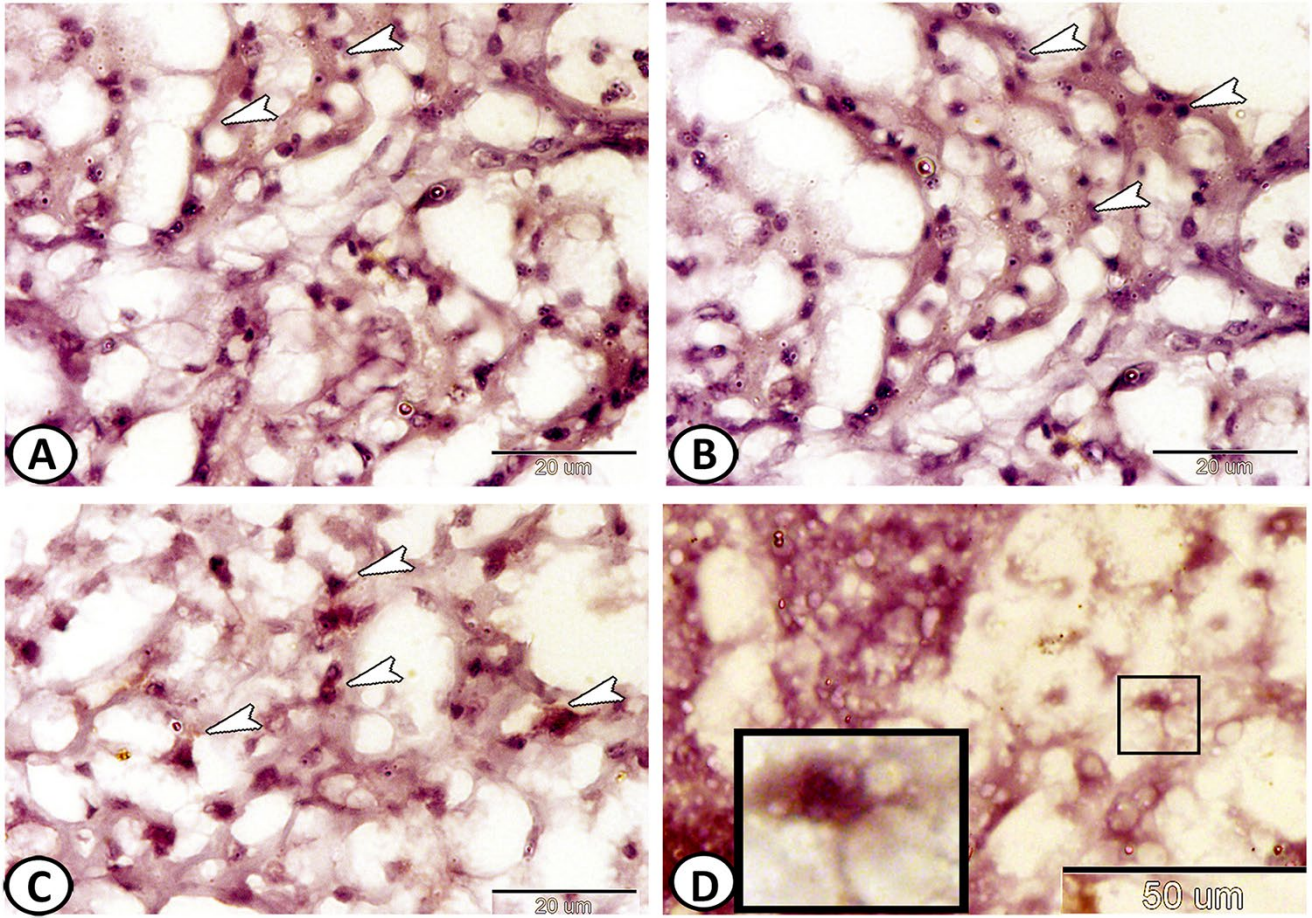
Immunohistochemical expressions of CD3 and CD8 in the pseudobranch. (**A**,**B**) PSCs expressed CD3 (arrowheads). (**C**) T-lymphocytes expressed CD3 (arrowheads). (**D**) Dendritic cells expressed CD8 (boxed areas).

**Figure 4:**
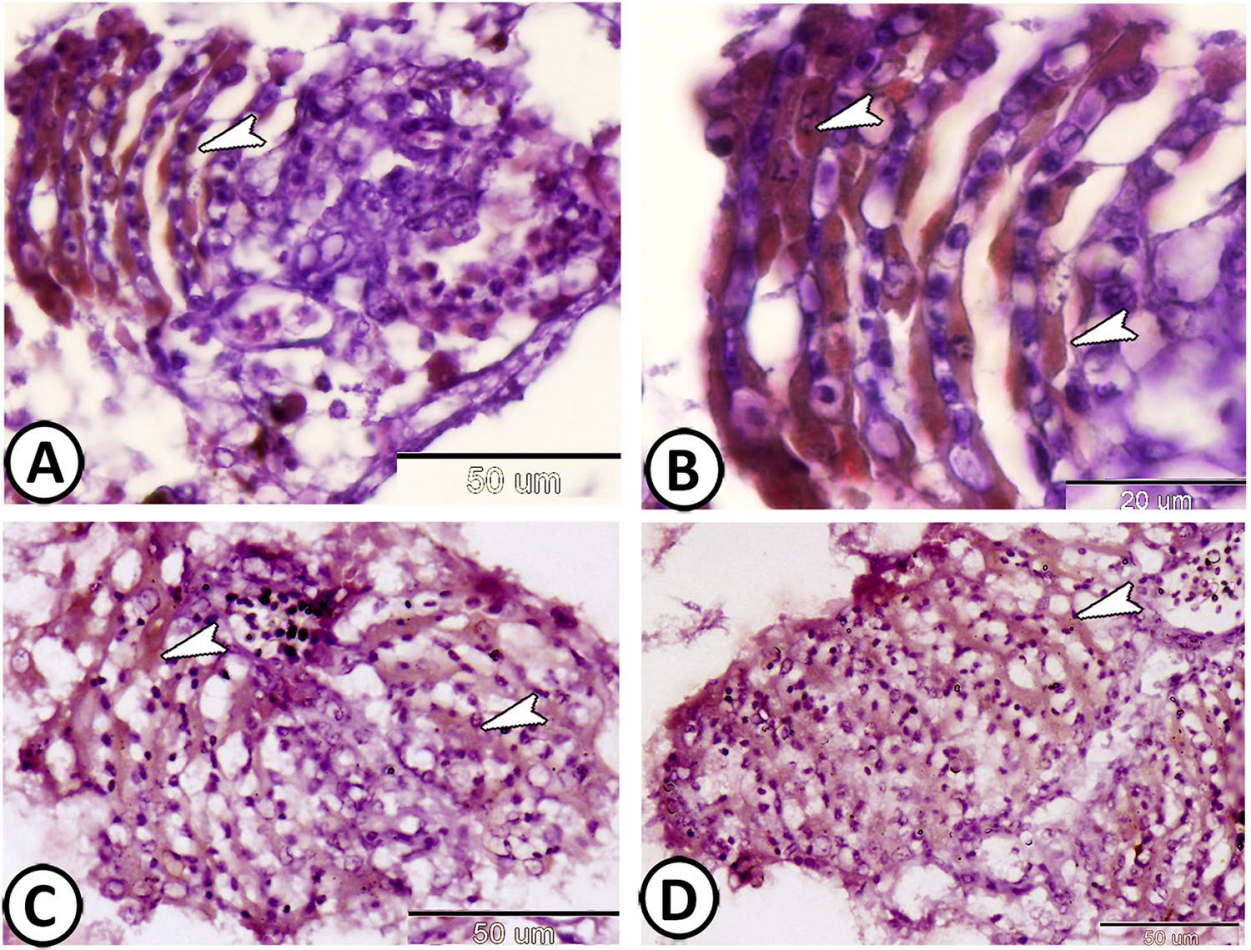
Immunohistochemical expressions of NF-κB and INOS-2 in the pseudobranch. (**A**,**B**) The PSCs (arrowheads) showed strong immunoreactivity for NF-κB. (**C**,**D**) The PSCs (arrowheads) also expressed iNOS-2.

Immunohistochemistry showed that the PSCs expressed CD3 (Fig. 3A,B). Many T-lymphocytes in the surrounding connective tissue showed positive expression to CD3 (Fig. 3C). Also, dendritic cells with dendrite-like processes in the connective tissue showed immunoreactivity for CD8 (Fig. 3D). Moreover, PSCs showed strong immunoreactivity for NF-κB (Fig. 4A,B) and iNOS-2 (Fig. 4C,D).

**Figure 5:**
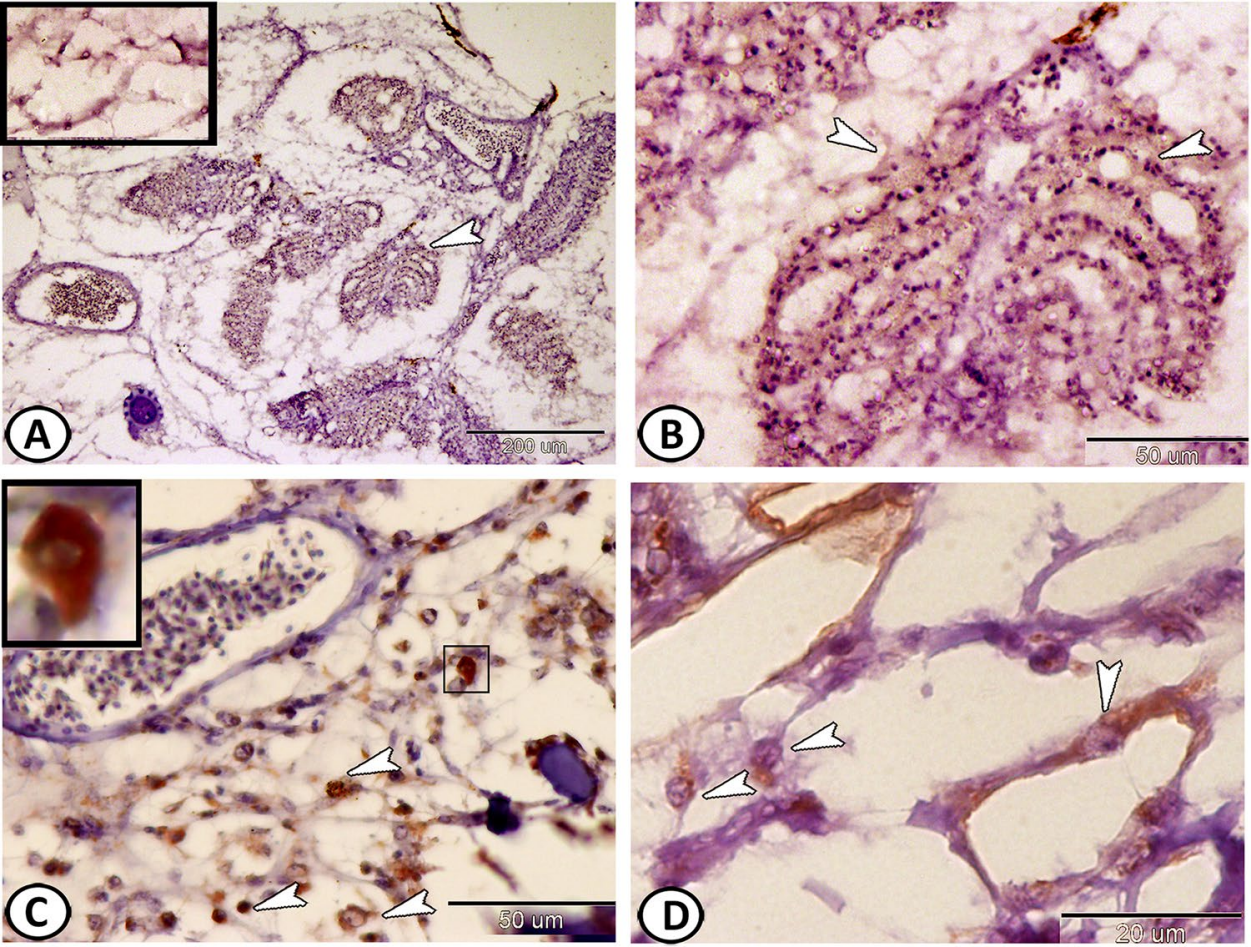
Immunohistochemical expressions of CD45 and APG-5 in the pseudobranch. (**A**,**B**) The PSCs (arrowheads) showed expressions for CD45. Note the distributed leucocytes in the connective tissue. (**C**,**D**) Macrophages (arrowheads) expressed APG-5.

PSCs also showed strong reactivity to CD45, as well as some leucocytes in the surrounding connective tissue could be observed (Fig. 5A,B). Many macrophage- positive APG-5 could be distinguished in the connective tissue surrounding the PSCs (Fig. 5C,D).

**Figure 6:**
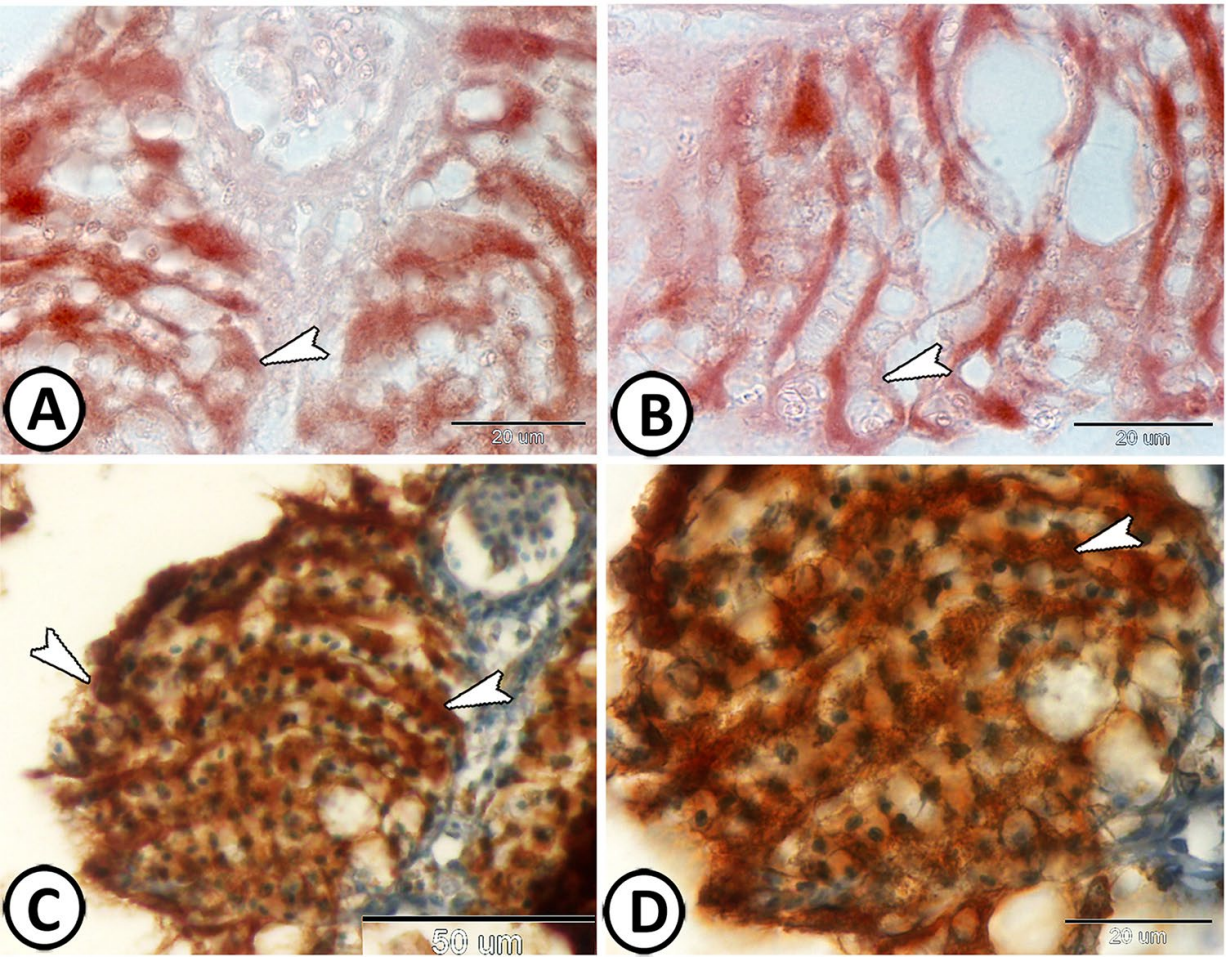
Immunohistochemical expressions of myogenin and TGF-β in the pseudobranch. (**A**,**B**) The PSCs (arrowheads) showed expressions for myogenin. (**C**,**D**) The PSCs (arrowheads) exhibited intense expressions for and TGF-β.

**Figure 7:**
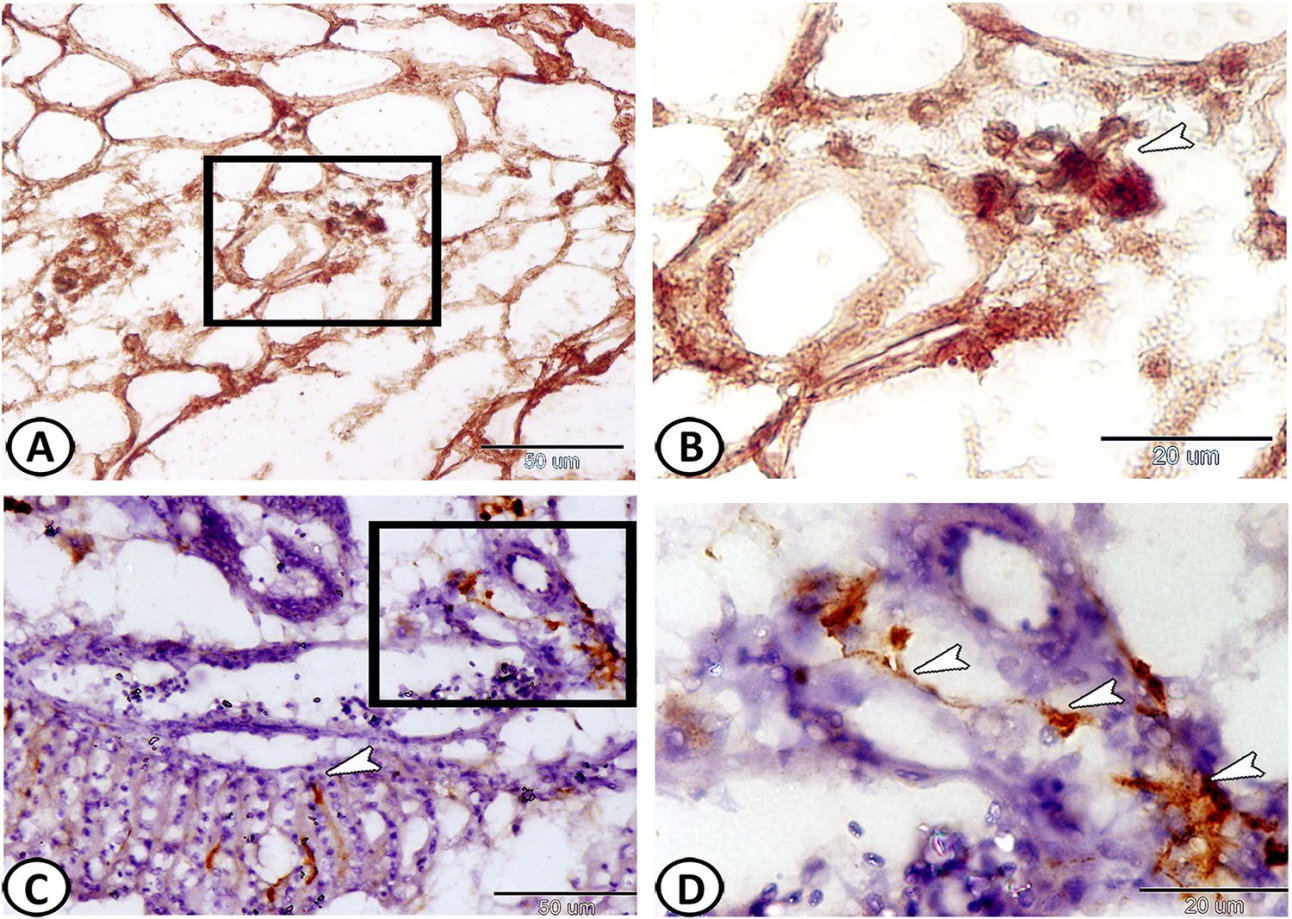
Immunohistochemical expressions of SOX-9 and GFAP. (**A**,**B**) stem cells expressed Sox-9 (boxed area, arrowhead). (**C**,**D**) GFAP- positive astrocytes (arrowheads) are observed in the surrounding connective tissue.

On the other hand, PSCs exhibited intense expressions of both myogenin (Fig. 6A,B) and TGF-β (Fig. 6C,D). In addition, SOX9- positive stem cells (Fig. 7A,B), as well as GFAP- positive astrocytes could be identified in the connective tissue surrounding it (Fig. 7C,D).

**Figure 8:**
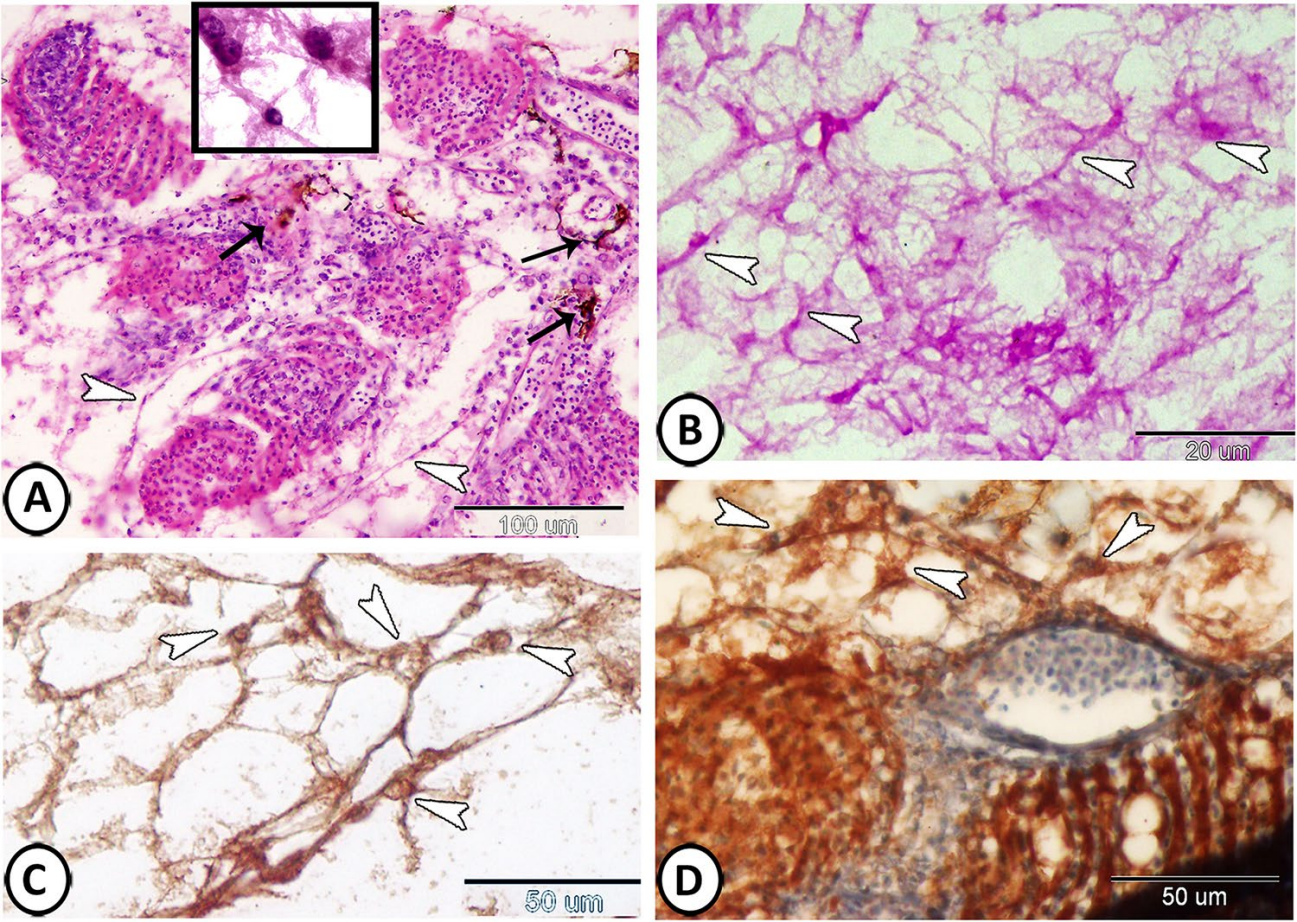
The distribution of telocytes (TCs) in the pseudobranch. (**A**) Many TCs (arrowheads, boxed areas) are demonstrated in the connective tissue surrounding the lamellae. Note the presence of many melanocytes (arrows) in the connective tissue around the pseudobranch. (HE). (**B**) The cell processes of TCs (arrowheads) established a network and stained positive with PAS. (**C**,**D**) TCs (arrowheads) expressed SOX9 and TGF-β respectively.

Many small, branched cells called telocytes (TCs) were demonstrated in the connective tissue surrounding the lamellae that were formed of a cell body containing a nucleus and many delicate cell processes (Fig. 8A). Their cell processes established a network and stained positive with PAS (Fig. 8B). Furthermore, TCs expressed SOX9 and TGF- β (Fig. 8C,D). Many melanocytes were also recognized in the connective tissue around the pseudobranch (Fig. 8A).

### Scanning electron microscopy (SEM)

**Figure 9:**
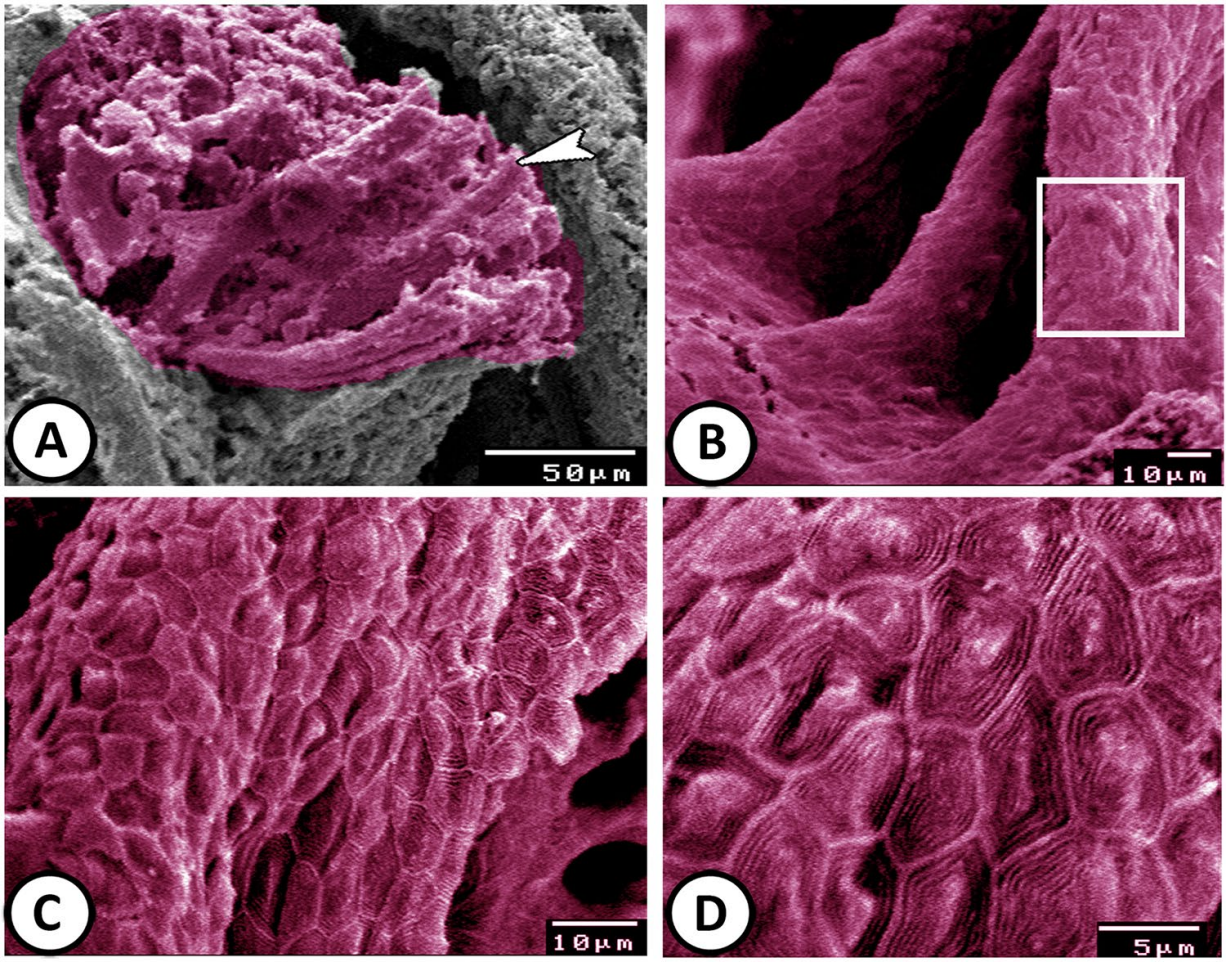
SEM of the pseudobranch. (**A**,**B**) The pseudobranch (arrowhead) of Molly fish is embedded in type and the lamellae are completely fused. (**C**) A magnified view of the boxed area in (**B**) showing that PSCs exhibit pentagonal or hexagonal shapes with well distinct borders. (**D**) The apical surface of these cells showed fingerprint-like patterns of microridges.

The pseudobranch of Molly fish was embedded in type and the lamellae were completely fused (Fig. 9A). There was no covering area of mucus (Fig. 9B). The surface view of the PSCs exhibited a pentagonal or hexagonal shape with well distinct borders (Fig. 9C). The apical surface of these cells showed fingerprint-like patterns of microridges (Fig. 9D).

## Discussion

The current results revealed that the Molly fish possessed an embedded type pseudobranch. The microscopic structure of the pseudobranch differs among the species of fish. The pseudobranch in fishes could be categorized into four classes based on its morphology; I) lamellae free type: These with distinct lamellae in contact with the water and the secondary lamellae are completely free such as the milkfish (*Chanos chanos*)^16,17^; II) Lamellae semi-free type: Those characterized by partially free lamellae and distinguishable filaments covered by the opercular membrane, such as silver moony (*Monodactylus argenteus*)^18^; III) Covered type: the pseudobranch is completely reduced and is covered with the opercular membrane and separated from the opercular chamber by the folds of connective tissue, such as those in rainbow trout (*Oncorhynchus mykiss*)^19^; and (IV) Embedded (glandular) type: pseudobranchs are small, and are embedded in the connective tissues with a stereoscopic arrangement of filaments, such as those in Mozambique tilapia (*Oreochromis mossambicus*)^18^. The lamellae-free and semi-free type pseudobranchs are involved in hypo-osmoregulation. Whereas the embedded and covered types have a potential role in vision or ion transport^1^.

In both semi-free and lamellae-free types, the pseudobranch epithelium possesses both chloride cells (CCs) and the pseudobranch cells (PSCs); but, in the embedded and covered types, only PSCs are present^4^. Generally, the PSCs are similar to branchial CCs in which their cytoplasm is packed with numerous mitochondria and an extensive tubular system^19^. Moreover, PSCs exhibit high Na^+^, K^+^-ATPase (NKA) activity in Chinook salmon (*Oncorhynchus tshawytscha*)^1^, milkfish^16^, silver moony, and Mozambique tilapia^18^.

The glandular structure of the pseudobranch of Molly fish with the presence of PAS-positive PSCs suggesting that the pseudobranch has a potential role in the secretion or storing of neutral mucopolysaccharides. Moreover, Laurent and Dunel-Erb^6^ proposed the release of carbonic anhydrase from the pseudobranch that was necessary for the respiratory transport of gases to the non-vascularized retina of some fish species.

On the other hand, the current results revealed the presence of many melanocytes in the connective tissue surrounding the pseudobranch. Roy et al.^20^ reported that in fish where the pseudobranch was removed, dark body coloration was assumed due to chromatophore expansion, and injections of pseudobranch extract led to the temporary paling of the body. Lange^21^ suggested that serotonin could be produced in the pseudobranchial cells and transported to the brain via the choroid rete, where it may have affected the *Corpus pineale*, which is responsible for the melatonin synthesis. The absence of chloride cells in the pseudobranch of Molly fish provides good evidence that the PSCs can react to environmental salinity and have a role in osmoregulation. SEM revealed that the PSCs were covered by microridges that may play a role in the increase of the surface area, which is important in ionic exchange.

The present study showed that the pseudobranch of Molly fish expressed NF- κB and iNOS-2. NF-κB is a key responder to immune and inflammatory stimuli and regulator of cell proliferation, apoptosis, and angiogenesis in several cell types^22^. Previous studies have reported the significant roles of NF-κB signaling for the immune homeostasis maintenance in barrier tissues^23^. Nitric oxide has been suggested to play a dual role in regulating immune responses^24^. It is a pro-inflammatory produced by iNOS in macrophages and other innate immune cells, and is considered an essential component of the host immune response against various pathogens^25^. Previous studies have reported the expression of iNOS by macrophages, T cells, and mature dendritic cells. These studies demonstrated that iNOS regulates immune cells differentiation and function through nitration of essential molecules involved in transcriptional or signaling pathways^26,27^. Furthermore, iNOS upregulates Na^+^ and HCO transport, which indicates its function in the acid–base balance^28^.

Macrophage- positive APG-5 could be distinguished in the connective tissue surrounding the PSCs. Autophagy-related gene 5 (APG5 or ATG5) is one of the essential regulators of the autophagy process^29^, where it is critical for the formation of autophagic vesicles, in addition to the development and proliferation of lymphocyte^30^. In addition, many other functions of ATG5 have been reported, including mitochondrial quality control following oxidative damage; negative regulation of the innate antiviral immune response, adipocyte differentiation; MHC II antigen presentation, and apoptosis^30^. A previous study on the grass carp revealed a higher expression of ATG5 in the gill and intestine, suggesting an important role of ATG12 in innate immunity and autophagy^31^.

Moreover, PSCs demonstrated strong immunoreactivity for TGF-β, which is a pleiotropic cytokine produced by immune and non-hematopoietic cells, and plays critical roles in oncogenesis, immune response suppression, cell proliferation, as well as modulation of ion transport^32,33^ through direct trafficking of the epithelial sodium channel^34^. Furthermore, PSCs expressed myogenin that is normally expressed in muscle cells, and is an essential regulator of muscle stem cell homeostasis and adult muscle fiber growth^35,36^. The presence of myogenin expression here may be an indicator of proliferation and regeneration functions of the pseudobranch. These findings have been supported by the extensive distribution of the TGF-β- positive telocytes, which their main function is organ regeneration^8^.

The current study revealed the presence of SOX9- positive stem cells and telocytes, in addition to GFAP- positive astrocytes in the pseudobranch of Molly fish. SOX family is important for stem cell maintenance^37^. SOX9 is a member of the SOX family, which has a critical role in the cell proliferation and cell fate regulation during embryogenesis^38–40^, in addition to its role in the regulation of stem and progenitor cells in adult tissues^37,41^. Previous studies proposed that the inhibition of SOX9 affects the regulation of extracellular matrix balance, and promotes the inflammatory and the immune responses^42^. GFAP is a major constituent of glial intermediary filaments that form the cytoskeleton of mature astrocytes, and is responsible for maintaining glial cells mechanical strength, and supporting neighboring neurons and the blood brain barrier^43,44^. The increased GFAP expression has been reported in inflammatory diseases of the gut^45^, and has been promoted in enteric glia by proinflammatory cytokines^46^.

The PSCs showed immunoreactivity for CD3 and CD45. T-lymphocytes in the surrounding connective tissue also expressed CD3. CD3 is an essential component of the CD3-T cell receptor complex, and is involved in immune responses^47^. The CD3 protein complex was first identified by immunoprecipitation techniques using human T cells^48^, and has been considered an essential T cell marker for the classification of T cell neoplasms. Moreover, CD3 has been used for the identification of T cells in various inflammatory disorders^49–51^. CD3 gene deficiency has been reported one of the causative agents of severe combined immune deficiency that is characterized by a massive defect in T cell production or function^52–54^.

CD45 is a transmembrane glycoprotein with tyrosine phosphatase activity, expressed on all nucleated hematopoietic cells except erythrocytes and platelets, with its eight isoforms are distributed through the immune system according to cell type and cellular differentiation degree^55,56^. It is one of the most abundant proteins within the T cell plasma membrane and is necessary for T cell antigen receptor signaling^57^. Within the T cell plasma membrane, the segregation of CD45 has been thought to be necessary for T cell activation^58^, as it is necessary for both the activation and suppression of T cells upon interaction with antigen-presenting cells^59^.

Dendritic cells (DCs) elucidated immunoreactivity for CD8. These cells originate in the bone marrow from macrophage/DC progenitors^60,61^, and are categorized as a professional antigen presenting cells found within a wide variety of tissues. They play a central role in linking innate and adaptive immune responses, initiating effectively all adaptive immune responses by uptaking, processing and presenting antigens to activate naive antigen-specific CD4 and CD8 T cells^62–64^.

## Material and methods

The present work was performed in accordance with the Egyptian laws and University guidelines for animal care. The National Ethical Committee of the Faculty of Veterinary Medicine, Assiut University, Egypt, has approved all the procedures in this study, which was also performed in accordance with ARRIVE guidelines.

### Sample collection

Randomly obtained 14 adult males of Molly fish (*Poecilia sphenops*) were used in this study and were purchased from ornamental fish shops. The anthropometric features of specimens were 4.20 ± 4.0 cm standard length and 10.60 ± 1.70 gm body weight.

### Histological and histochemical analysis

Pseudobranchs were dissected as soon as possible and were embedded in Bouin’s fixative solution for 22 h. The fixed samples were processed for light microscopy analysis through dehydration with ethanol, clearance by methyl benzoate, and embedding in paraffin wax. Serial sagittal and transverse paraffin sections of about 5 μm thickness were cut and stained with various histochemical stains including Harris hematoxylin and Eosin, PAS, Crossmon' trichrome, and Giemsa's stain. These stains were performed according to Bancroft and Gamble^65^.

### Immunohistochemical analysis

Sections of the pseudobranch were prepared for immunohistochemical analysis using Pierce Peroxidase Detection Kit (36000, Thermo Fisher Scientific, UK). The sections were then processed following the instruction of the kit as described previously^66^. The sections were incubated overnight at 4 °C with diluted primary antibodies against monoclonal anti-mouse transforming growth factor beta (TGf-β) (1:100, MA5-16949, Thermo Fischer Scientific, UK), nuclear factor kappa B (NF-κB) (10745-1-AP, Proteintech, USA), glial fibrillary acidic protein (GFAP) (PA5-16291, Thermo Fisher Scientific, Waltham, USA), autophagy protein 5 (APG5) (sc-133158, Santa Cruz Biotechnology, Heidelberg, Germany), a rabbit polyclonal anti-CD3 (cluster of differentiation 3) (1:200; Abcam, Cambridge, UK, ab828), mouse polyclonal anti-CD8 (1:200; Abcam, Cambridge, UK), a rabbit polyclonal antibody inducible nitric oxide synthase (iNOS-2) (RB-1605, Thermo Fisher Scientific, UK), mouse monoclonal CD45 (1:100, 14-0454-82, Thermo Fischer Scientific, UK), myogenin (AB3239, Sigma-Aldrich, Madrid, Spain), and SRY-Box transcription factor 9 (Sox9) (AB5535, Sigma-Aldrich, Madrid, Spain),SRY-Box transcription factor 9 (Sox9). The slides were then incubated with diluted (1:1000) goat anti-rabbit IgG (65–6140, Invitrogen, USA) and diluted (1:100) goat anti-mouse IgG (31800, Invitrogen, USA) secondary antibodies. Following that, the slides were washed, incubated with the diluted (1:500) Avidin-HRP (43-4423, Invitrogen, USA), followed by incubation with 1X metal-enhanced DAB substrate working solution. Finally, the sections were counterstained with Harris modified hematoxylin. As a negative control, experiments were performed without the primary antibodies. Table 1 summarizes the information of the used first antibodies.

**Table 1:** Suppliers and dilutions of the first antibodies used.

Antibody	Supplier	Dilution
Transforming growth factor beta (TGf-β)	Santa Cruz Biotechnology, Heidelberg Germany	1:100
Nuclear factor kappa B (NF-κB)	Santa Cruz Biotechnology, Heidelberg Germany	1:100
Glial fibrillary acidic protein (GFAP)	Thermo Fisher Scientific, UK	1:100
Autophagy protein 5 (APG5)	Santa Cruz Biotechnology, Heidelberg Germany	1:100
Rabbit polyclonal anti- CD3	Abcam, Cambridge, UK	1:200
Mouse polyclonal anti-CD8	Abcam, Cambridge, UK	1:200
Inducible nitric oxide synthase (iNOS-2)	Thermo Fisher Scientific, UK	1:100
Mouse monoclonal CD45	Thermo Fischer Scientific, UK	1:100
SRY-Box transcription factor 9 (Sox9)	Sigma-Aldrich, Madrid, Spain	1:100
Myogenin	Santa Cruz Biotechnology, Heidelberg Germany	1:100

### Scanning electron microscopy

Small pseudobranch specimens were washed in sodium phosphate buffer (0.1 M) and were fixed in a mixture of 2.5% paraformaldehyde and 5% glutaraldehyde in the same buffer, at 4 °C for 24 h. The samples were then post-fixed in 1% osmic acid for two hrs. at room temperature, followed by dehydration using acetone. The specimens were exposed to critical-point drying using a Polaron apparatus and were coated with gold using JEOL- 1100 E ion sputtering device. Finally, the samples were examined with a JEOL scanning electron microscope (JSM 5400 LV) at 10 kV.

## Conclusion

This study highlighted for the first time the structure and potential functions of the pseudobranch in Molly fish. The presence of PAS-positive PSCs suggests the secretory or storage properties of pseudobranch. The immunoreactivity of pseudobranch for NF- κB and iNOS-2 supports its role in the regulation of immune responses and cell proliferation, in addition to the maintenance of immune homeostasis and acid–base balance; meanwhile, the presence of macrophage- positive APG-5 proposes the potential role of PSCs in autophagy, and lymphocyte development and proliferation. Furthermore, strong immunoexpression of PSCs for TGF-β indicates critical roles of pseudobranch in immune response suppression, cell proliferation, and ion transport modulation, while high immunoreactivity for myogenin may be an indicator of proliferation and regeneration functions of the pseudobranch. The presence of SOX9- positive stem cells and GFAP- positive astrocytes indicates role of pseudobranch in stem cell maintenance. The expression of PSCs for CD3 and CD45 supports their role in T cell activation; meanwhile, the immunoreactivity of dendritic cells for CD8 indicates the role of pseudobranch in the regulation of adaptive immune responses. Accordingly, the present findings confirm the potential roles of pseudobranch of Molly fish in immunity and cell regeneration. Further functional and molecular studies should be performed to assure its potential roles in immune system.

## Data Availability

The datasets analyzed during this study are available from the corresponding author on reasonable request.
